# Melatonin in Assisted Reproductive Technology: A Pilot Double-Blind Randomized Placebo-Controlled Clinical Trial

**DOI:** 10.3389/fendo.2018.00545

**Published:** 2018-09-19

**Authors:** Shavi Fernando, Euan Morrison Wallace, Beverley Vollenhoven, Nicholas Lolatgis, Nicole Hope, Melissa Wong, Mark Lawrence, Anthony Lawrence, Chris Russell, Kenneth Leong, Philip Thomas, Luk Rombauts

**Affiliations:** ^1^Department of Obstetrics and Gynaecology, Monash University, Clayton, VIC, Australia; ^2^The Ritchie Centre, Hudson Institute of Medical Research, Clayton, VIC, Australia; ^3^Monash IVF, Richmond, VIC, Australia

**Keywords:** melatonin, Assisted Reproductive Technology, ART, *in-vitro* fertilization, IVF, clinical pregnancy

## Abstract

**Purpose:** To explore in a small pilot study whether oral melatonin, administered during ovarian stimulation increases clinical pregnancy rate (CPR) after IVF and what dose might be most effective.

**Methods:** Pilot double-blind, dose-finding, placebo-controlled randomized clinical trial in private IVF clinics in Australia between September 2014 and September 2016. One hundred and sixty women having their first cycle of IVF or ICSI were randomized to receive placebo (n = 40), melatonin 2 mg (n = 41), melatonin 4 mg (n = 39), or melatonin 8 mg (n = 40) twice per day (BD) during ovarian stimulation. The primary outcome was CPR. Secondary outcomes included serum and follicular fluid (FF) melatonin concentrations, oocyte/embryo quantity/quality, and live birth rate (LBR). Analysis was performed using the intention-to-treat principle.

**Results:** There was no difference in CPR or LBR between any of the four groups (p = 0.5). When all the doses of melatonin were compared as a group with placebo, the CPR was 21.7% for the former and 15.0% for the latter [OR 1.57 (95% CI 0.59, 4.14), p = 0.4]. There were also no differences between the groups in total oocyte number, number of MII oocytes, number of fertilized oocytes, or the number or quality of embryos between the groups. This is despite mean FF melatonin concentration in the highest dose group (8 mg BD) being nine-fold higher compared with placebo (P < 0.001).

**Conclusion:** No significant differences were observed in CPR or oocyte and embryo parameters despite finding a nine-fold increase in FF melatonin concentration. However, this study was not sufficiently powered to assess differences in CPR and therefore, these results should be interpreted with caution. Because this was a small RCT, a beneficial effect of melatonin on IVF pregnancy rates cannot be excluded and merits confirmation in further, larger clinical trials. ANZCTR (http://www.anzctr.org.au/ Project ID: ACTRN12613001317785).

## Introduction

Improving the success rates of IVF remains a focus of infertility research. Recently, interest has grown in the effects of oxidative stress on the success rates of ART. ART exposes oocytes and embryos to high concentrations of reactive oxygen species (ROS) during gamete and embryo culture (1). Oxidative stress in the follicular fluid (FF) of infertile women are associated with poor oocyte maturation and embryo quality, and inducers of oxidative stress inhibit oocyte maturation (2, 3). It has been suggested that anti-oxidant therapy might lessen detrimental effects of excessive ROS and so improve success rates (4).

Melatonin can mitigate oxidative stress by neutralizing ROS in human tissues and by inducing endogenous anti-oxidant enzymes (5). Melatonin has been suggested as an adjuvant therapy in the management of diverse medical conditions in which oxidative stress has been implicated, including diabetes, glaucoma, irritable bowel syndrome, and infertility (6–9). That melatonin receptors are found on granulosa cells, oocytes, and embryos (10), suggests that it may have a physiological role in reproduction(2–5, 11, 12). What that role is, if any, and whether it relates to anti-oxidant protection remains unknown. Nonetheless, it is intriguing that higher concentrations of melatonin in the ovarian follicle are associated with higher follicular progesterone and lower estradiol concentrations (13).

Several human and animal studies support the use of melatonin in the management of infertility (2, 4, 11, 12, 14). Beneficial effects, if any, have been largely attributed to its oxygen scavenging properties (5). However, clinical trials addressing the use of melatonin in IVF have been small, lack blinding and were not placebo-controlled (2, 4, 11, 12). There have also been no attempts to define an optimal dosing regimen (15). Considering the short half-life of melatonin (16), a single daily dose may not provide sustained protection from oxidative stress. Further, interpretation of trial outcomes have been hampered by poor study design, where participants have acted as their own controls (2, 12, 17).

The lack of existing data and the absence of previous multiple-dose testing studies prompted us to undertake a pilot study with a randomized, double-blinded, placebo-controlled dose-finding trial design to investigate the effect(s), if any, of oral melatonin on the clinical pregnancy rate and other secondary outcome measures following IVF/ICSI treatment. The aim of our study was to (a) estimate the sample size and optimal melatonin dose needed for a future trial, (b) examine the plausibility of the effectiveness of the intervention through the measurement of secondary outcomes, including follicular fluid concentrations of melatonin and quantity/quality of oocytes and embryos, (c) assess the recruitment rate, trial adherence and retention, and (d) record adverse maternal and fetal outcomes of twice daily high dose melatonin administration (18).

## Materials and methods

### Participants

Women were recruited at their first clinical contact visit. Baseline demographic data obtained included age, BMI, smoking status, parity, ethnicity, etiology of infertility, and night shift working status.

Women were eligible if they (i) were undergoing their first cycle of IVF/ICSI, (ii) were undergoing an antagonist cycle, (iii) were aged between 18 and 45, and (iv) had a BMI between 18 and 35. Exclusion criteria were: untreated endometriosis, uterine malformations, large distorting fibroids or endometrial polyps, autoimmune disease, concurrent use of other adjuvant therapies, malignancy, preimplantation genetic screening, known sensitivity to melatonin, or if concurrently taking medications known to interact with melatonin (antidepressants, antiepileptics, or hypnotics) (19).

### Recruitment

**Figure 1:**
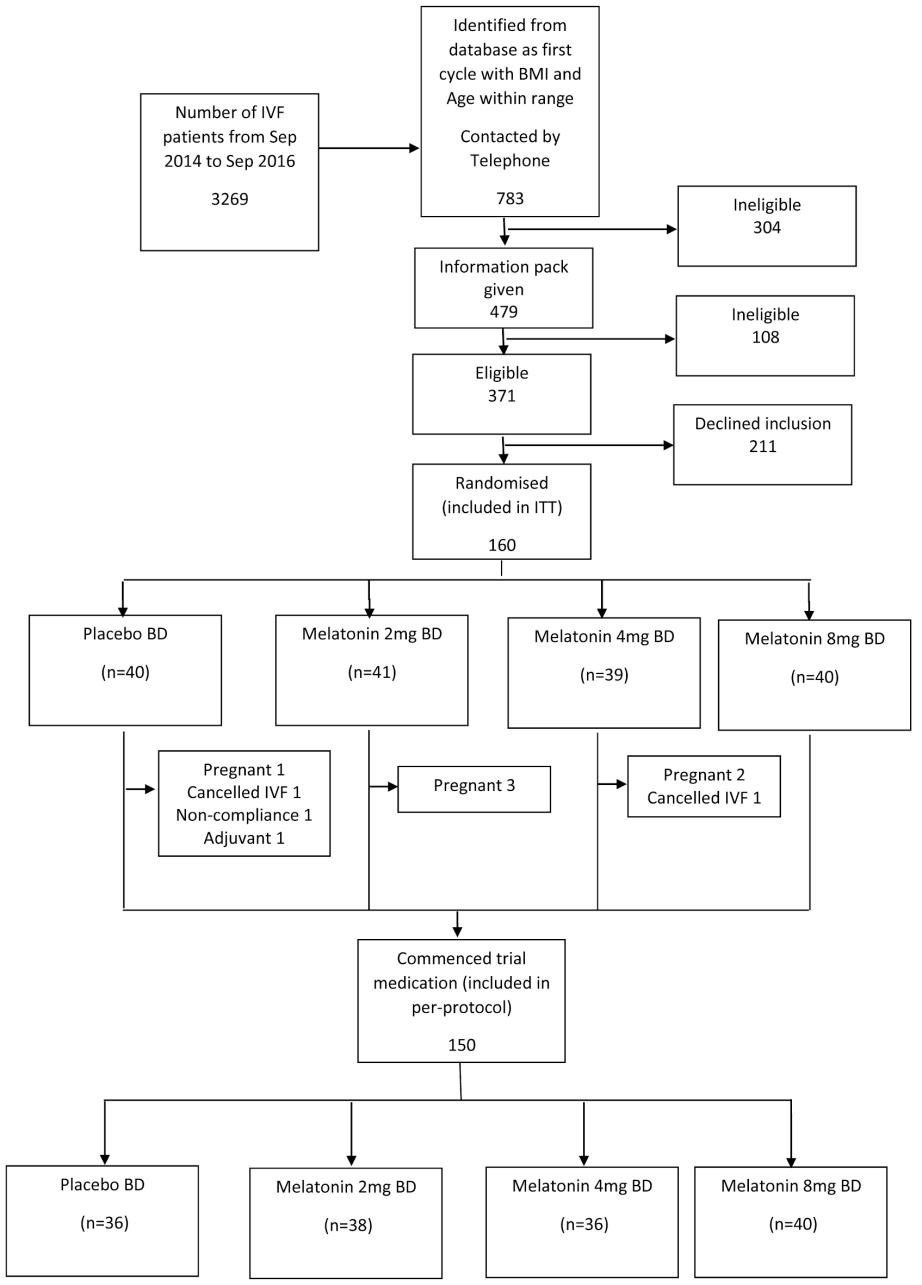
Recruitment flowchart. BD, twice per day; ITT, intention-to-treat; IVF, *in-vitro* fertilization.

We planned to recruit 160 women, 40 in each group. Three thousand two hundred and sixty-nine women underwent their first cycle of IVF/ICSI at Monash IVF between September 2014 and September 2016. Following several steps of eligibility assessment, 371 were found to be eligible, of which 211 declined to participate, leaving 160 to be randomized (Figure 1). Ten (6.3%) of these women were withdrawn after randomization but before commencement of trial medication because they subsequently met exclusion criteria (pregnant before trial medication, n = 6; used excluded adjuvants, n = 1; canceled IVF, n = 2; could not comply with trial protocol, n = 1), leaving a total of 150 women (Figure 1).

### Blinding and randomization

Each dose of trial medication was randomly allocated a letter designation (“A” to “D”) using a random number generator by the trial's independent Data Safety and Monitoring Board (DSMB). This allocation was only known to the DSMB and the hospital pharmacy responsible for labeling and dispensing the medication until after completion of the trial. All trial investigators, clinicians, and participants remained blinded throughout the trial. All medication bottles and capsules were of identical appearance.

To prevent selection bias, randomization was performed using the minimization method, accounting for factors known to affect the outcome used in small trials (20). Weighted minimization was performed using age (weighting of 20), parity (weighting of 10), BMI (weighting of 5), and smoking status (weighting of 1) in real-time at enrolment using minimization software (MUI Online Minimization®, powered by Qminim®).

### Outcome measures

The primary outcome was clinical pregnancy rate (presence of a live intrauterine pregnancy detected on transvaginal ultrasound scan at 6–8 weeks' gestation). The secondary outcomes were live birth rate, oocyte and embryo number, and quality, number of oocytes fertilized, number of embryos utilized, rates of miscarriage, pregnancy complications, and adverse events including cycle cancelations (19).

### Administration of trial medication and compliance

**Figure 2:**
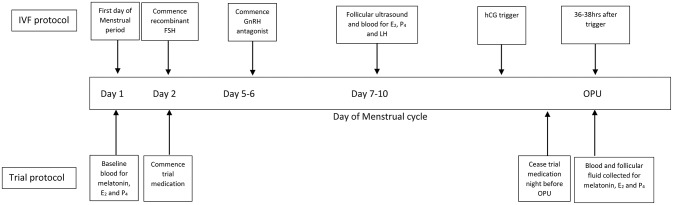
IVF and general trial protocol. IVF, *in-vitro* fertilization; FSH, follicle stimulating hormone; GnRH, gonadotrophin releasing hormone; hCG, human chorionic gonadotrophin; E_2_, estradiol; P_4_, progesterone; OPU, oocyte retrieval.

All active formulations of trial medication were “sustained release” (containing melatonin derived from Diethyl Malonate and Acrylonitrile, 40% methocel E4M and methylcellulose encased in a gelatine capsule). Trial medication was specifically manufactured to appear the same by Orrong Compounding Pharmacy, Melbourne, Australia. The melatonin content of all formulations was independently verified using High Performance Liquid Chromatography against a melatonin reference standard (Melatonin Fagron B#13D09-U03-010769, Purity 99.4%) (Australian Life Sciences, NSW, Australia). Placebo capsules were composed of methylcellulose. Each participant was instructed to take one capsule twice per day (once between 08:00 and 10:00 and once between 20:00 and 22:00) from day 2 of their cycle until the night before oocyte retrieval (Figure 2). Each participant also kept a diary documenting compliance and adverse events.

### Ovarian stimulation and oocyte retrieval (OPU) protocol

All patients received a standard fixed GnRH antagonist protocol with recombinant FSH for ovarian stimulation with a starting dose (150–450 IU) decided by the treating clinician. A recombinant HCG trigger (250 or 500 IU depending on body weight) was administered 36 h before their OPU (Figure 2). Cycles were canceled if there were fewer than three follicles >17 mm or if there was risk of severe OHSS. Transvaginal ultrasound-guided OPU was performed under general anesthetic.

### Collection of blood

Blood was collected to assess melatonin, estradiol (E2), and progesterone (P4) concentrations on the day of recruitment, on D8-9 of ovarian stimulation and on the day of OPU (prior to general anesthetic). Blood was transported in light-shielded containers, centrifuged at 1,800 g at 21°C for 15 min and frozen in aliquots at −80°C.

### Collection of FF

At OPU, FF was collected from the single largest, most accessible follicle. The volume of collection media (Sydney IVF Follicle Flush Buffer, Cook Medical Australia) was standardized to allow for accurate concentration comparisons between samples. Following oocyte retrieval, samples were immediately transported on ice in shielded containers and centrifuged at 400 g at 4°C for 7 min to remove cellular debris and aliquoted at −80°C.

### Melatonin assay

Serum melatonin concentrations were determined by radio-immunoassay (Buhlmann®, RK-MEL2, Switzerland), according to manufacturer's instructions. The extraction efficiency of the assay was >90%, with an estimated functional sensitivity (CV = 10%) of 0.9 pg/ml and an estimated analytical sensitivity of 0.3 pg/ml.

### Serum progesterone and estradiol assay

Steroid concentrations were assayed with Chemiluminescent Microparticle Immunoassays (Architect iSystem, Illinois, USA) (progesterone sensitivity <0.1 ng/ml; estradiol sensitivity <10 pg/ml).

### FF progesterone and estradiol assay

estradiol concentration in FF was determined using mass spectrometry (AbSciex Triple Quad 5500 LC/MS/MS system) following solvent extraction. Progesterone was determined using a Beckman Coulter® competitive binding immunoenzymatic assay performed on a Unicel DXI® 800 analyzer (Lane Cove, Australia).

### Embryology procedures and assessments

Standard IVF or ICSI was performed using routine protocols. Experienced embryologists scored oocyte maturity and developing embryos for morphology on Day 3 and Day 5. Embryo quality was graded by blinded embryologists from “A” to “D.” “X” was used to describe zygotes that arrested before day 3. Good quality embryos were defined as those scored “A” or “B.” Embryo transfer occurred on day 3 or 5 and surplus embryos were frozen.

### Sample size and statistical analysis

There were no previously well-designed randomized placebo-controlled studies on which to base a power calculation for clinical pregnancy or live birth rate. Therefore, this was designed as a pilot study with a sample size of convenience (18). Ethnicity was grouped into Caucasian, Asian and South-Asian, and other. Where participants were canceled before their OPU, their number of oocytes and embryos were entered as zero.

All data were tested for normality using histograms. Analyses for all dichotomous categorical variables were performed using Chi-square tests or Fisher's exact test when cell values were <5. Normally distributed continuous data was analyzed across the four groups using ANOVA, otherwise the non-parametric equivalent was used (Kruskal–Wallis). Within-patient melatonin concentrations were analyzed with repeated-measures ANOVA using log-transformed data. Melatonin concentrations between groups were analyzed using ANOVA. Trends across dosing groups for categorical and continuous clinical outcomes were assessed using Chi-square for trend and Spearman's rank correlation coefficient respectively. Trends across groups for count variables were analyzed using Poisson regression. The primary outcome was also assessed using univariate and multivariate logistic regression. As planned a priori, prespecified subanalyses were performed by combining treatment groups and comparing outcomes with placebo using Mann–Whitney-U. To determine effects of canceled cycles, all primary outcome analyses were repeated after excluding these cases.

An independent interim analysis of safety data only (canceled cycle rate, miscarriage and medication side effects) was performed by the DSMB at 50% recruitment, with stopping rules defined prior to this assessment (19). Following this interim analysis, a recommendation to undergo a further interim analysis at 75% recruitment was requested by the DSMB due to a potential effect on the rate of canceled cycles. Following this second analysis, the DSMB approved the trial to be continued to completion.

Statistical analysis was performed using SPSS v22.0 (IBM, Armonk, New York). P < 0.05 was considered statistically significant for the primary outcome. For a priori determined secondary outcomes, we set the level of statistical significance at a more conservative level of p < 0.005 to control for multiple comparisons. ITT results have been reported for all primary and secondary outcomes unless otherwise stated.

### Ethics

This study was registered with the ANZCTR (Project ID: ACTRN12613001317785) with the protocol published before commencement of recruitment (19). Human research ethics approval was obtained from the Monash Health HREC (Project number: 13402B), Monash Surgical Private Hospital HREC (Project number: 14107), Monash University HREC (Project number: CF14/523–2014000181), and Epworth HealthCare HREC (Project number: 634-34). All participants gave written informed consent in accordance with the Declaration of Helsinki.

## Results

### Demographics

There were no differences at baseline for demographic variables (Table 1). Thirty women (19%) were aged ≥40 years and were evenly distributed across the groups. The 10 participants who withdrew did not differ from those that were included in the analysis.

**Table 1:** Demographics^a^.

**Treatment arm**	**Placebo** **N = 40**	**Melatonin 2 mg bd** **N = 41**	**Melatonin 4 mg bd** **N = 39**	**Melatonin 8 mg bd** **N = 40**	**Total** **N = 160**
Age Mean (SD)^b^	35.2 (4.2)	35.0 (4.1)	36.0 (4.2)	35.4 (4.4)	35.4 (4.2)
BMI Mean (SD)^b^	24.5 (4.8)	24.6 (4.0)	24.6 (4.5)	24.6 (3.9)	24.6 (4.3)
Gravidity 0, N (%)	22 (55.0)	24 (58.5)	26 (66.7)	24 (60.0)	96 (60.0)
Gravidity ≥1, N (%)	18 (45.0)	17 (41.5)	13 (33.3)	16 (40.0)	64 (40.0)
Parity 0, N (%)	32 (80.0)	35 (85.4)	33 (84.6)	32 (80.0)	132 (82.5)
Parity ≥1, N (%)	8 (20.0)	6 (14.6)	6 (15.4)	8 (20.0)	28 (17.5)
Current smoker N (%)	4 (10.0)	2 (4.9)	1 (2.6)	2 (5.0)	9 (5.6)
Night shift worker N (%)	1 (2.5)	1 (2.4)	2 (5.1)	3 (7.5)	7 (4.4)
TYPE N (%)^*^					
IVF	16 (40.0)	12 (29.3)	18 (46.2)	13 (32.5)	59 (36.9)
ICSI	24 (60.0)	25 (61.0)	21 (53.8)	26 (65.0)	96 (60.0)
Both IVF and ICSI	0 (0.0)	4 (9.8)	0 (0.0)	1 (2.5)	5 (3.1)
**ETHNICITY N (%)**					
Caucasian	20 (50.0)	30 (73.2)	28 (71.8)	28 (70.0)	106 (66.3)
Asian and South-Asian	14 (35.0)	9 (22.0)	9 (23.1)	7 (17.5)	39 (24.4)
Other	6 (15.0)	2 (4.9)	2 (5.1)	5 (12.5)	15 (9.4)
**ETIOLOGY N (%)**					
Endometriosis	6 (15.0)	7 (17.1)	5 (12.8)	5 (12.5)	23 (14.4)
PCOS	1 (2.5)	4 (9.8)	1 (2.6)	4 (10.0)	10 (6.3)
Tubal	7 (17.5)	8 (19.5)	4 (10.3)	5 (12.5)	24 (15.0)
Male factor	7 (17.5)	13 (31.7)	10 (25.6)	14 (35.0)	44 (27.5)
Ovulatory	1 (2.5)	1 (2.4)	0 (0.0)	1 (2.5)	3 (1.9)
Social^c^	1 (2.5)	2 (4.9)	4 (10.3)	2 (5.0)	9 (5.6)
Idiopathic	19 (47.5)	17 (41.5)	20 (51.3)	14 (35.0)	70 (43.8)

a Chi-square for all categorical variables, with Fisher's exact where necessary (cell value < 5).

b One-Way ANOVA.

c Social includes same-sex couples and single women.

* *These represent the number of women, at randomization, who were planned to receive either IVF, ICSI, or both IVF and ICSI*.

### Compliance

Compliance across groups was over 95%. The number of tablets and duration of intake did not differ between groups.

### Serum and FF melatonin concentrations

**Figure 3:**
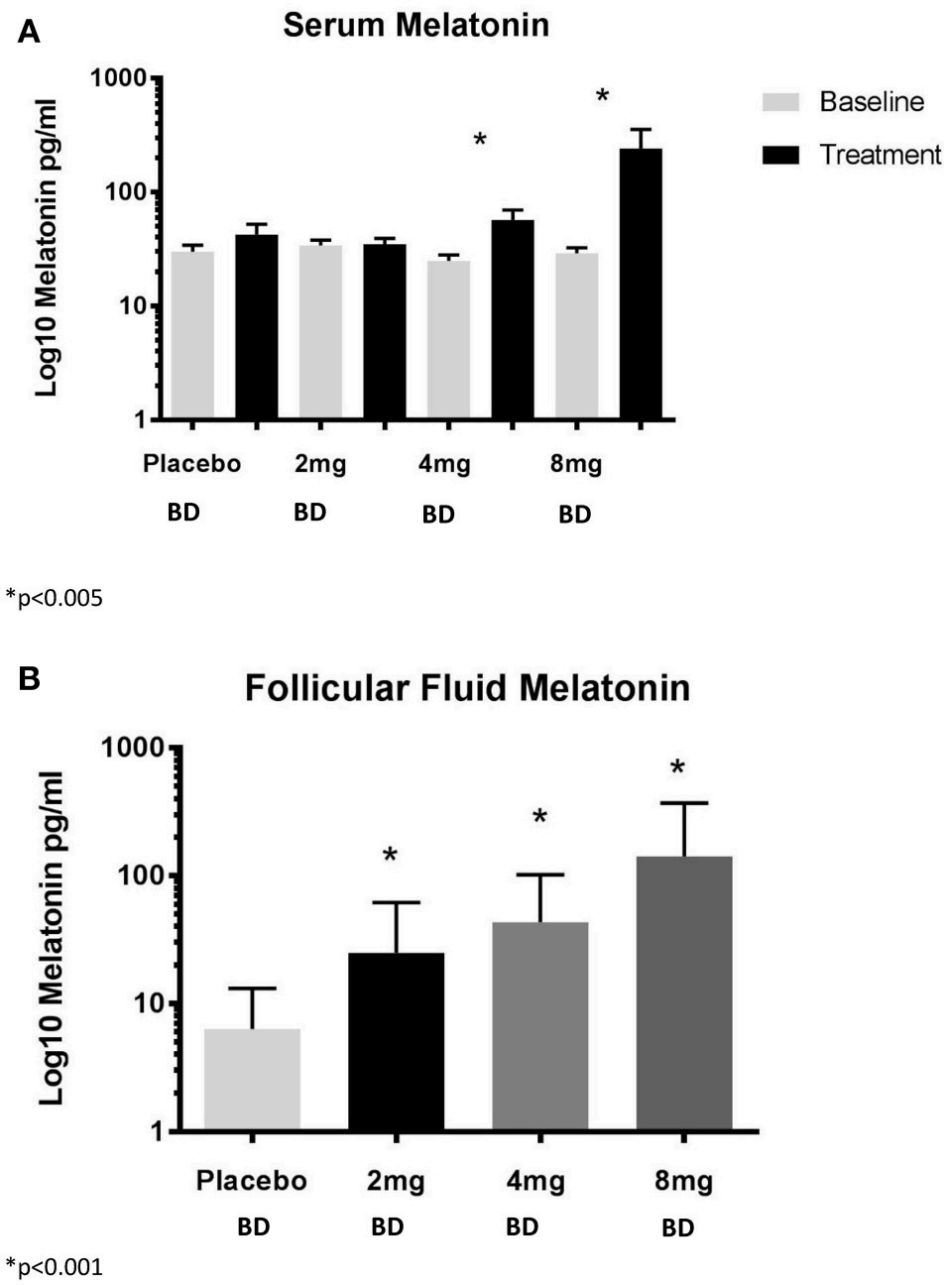
**(A)** Serum concentrations of melatonin and **(B)** Follicular fluid concentrations of melatonin. BD, twice per day.

OPU occurred 14.2 ± 1.7 h after the last dose of trial medication and increasing doses of melatonin resulted in increasing serum and FF concentrations of melatonin (Figures 3A,B).

### Serum estradiol and progesterone concentrations

There were no statistically significant differences or dose-dependent effect on day 8–9 serum concentrations of estradiol, progesterone or LH (Table 2) across all groups or when treatment groups were combined and compared with placebo. There was no statistically significant difference or dose-dependent effect on FF concentrations of estradiol and progesterone (Table 2).

**Table 2:** Sex-steroid concentrations at baseline, day 8–9 and at oocyte retrieval (Median, IQR).

	**Placebo** **N = 34**	**Melatonin** **2 mg bd** **N = 29**	**Melatonin** **4 mg bd** **N = 29**	**Melatonin** **8 mg bd** **N = 34**	**P-value^a^**	**P-value for trend across** **groups^b^**	**Any melatonin** **N = 92**	**P-value^c^**
**SERUM**								
**Baseline**								
Estradiol (pmol/L)	242 (152–414)	388 (189–612)	333 (162.5–464)	357 (225–549)	0.3	0.2	355 (187–524)	0.1
Progesterone (nmol/L)	11.6 (1.0–33.9)	19.8 (2.5–36.6)	11.9 (1.05–44.7)	23.7 (5.4–28.5)	0.7	0.5	18.1 (3.3–35.3)	0.4
**DAYS 8–9**								
Estradiol (pmol/L)	2,309 (1,310–3,079)	2,264 (984–4,231)	2,355 (1,193–4,456)	2,178 (1,051–3,666)	0.9	0.8	2249 (1,152–4,129)	0.8
Progesterone (nmol/L)	1.5 (0.93–2.18)	1.0 (0.8–1.6)	1.4 (0.9–2.5)	1.0 (0.7–1.9)	0.1	0.2	1.1 (0.8–2.0)	0.1
LH (IU/L)	1.2 (1.0–2.2)	1.4 (0.0–2.6)	1.6 (0.0–2.5)	1.0 (0.0–2.3)	0.8	0.4	1.3 (0.0–2.3)	0.6
**DAY OF OPU**								
Estradiol (pmol/L)	2,287 (1,755–3,045)	2,642 (1,705–3,447)	1819.5 (1592.5–3204.75)	1,985 (1,337–3040.3)	0.4	0.3	2,131 (1624.25–3276.75)	0.8
Progesterone (nmol/L)	23.75 (18.28–33.25)	22.0 (16.88–35.05)	21.6 (17.35–27.05)	21.35 (12.53–32.85)	0.8	0.3	21.7 (16.58–32.13)	0.4
**FOLLICULAR FLUID**								
Estradiol (pmol/L)	851,000 (529,500–1,290,000)	705,000 (425,000–1,190,000)	629,000 (377,000–1,115,000)	708,000 (517,000–1,180,000)	0.9	0.4	701,500 (425,000–1,177,500)	0.3
Progesterone (nmol/L)	25,808 (18,841–32,937)	22,166 (15,293–33,636)	25,028 (18,367–30,369)	30,449 (14,865–35,466)	0.6	0.8	25,037 (16,950–33,872)	0.7

a Kruskal–Wallis.

b Spearman-rank correlation.

c *Mann–Whitney U; Column totals are based on the number of samples which were analyzed*.

### Oocyte number and maturity and embryo number and quality

There was no statistically significant difference between the groups for total oocyte number (p = 0.8) or for the number of oocytes that were fertilized (p = 0.6). There was also no significant dose-dependent trend in any oocyte or embryo parameter (Table 3). There was also no significant difference in the proportion of good quality embryos transferred between placebo and melatonin groups (p = 0.5). When assessing the number of MII oocytes (data was available for ICSI patients, N = 96), there was no significant difference in the median number of MII oocytes between the groups (p = 0.4).

**Table 3:** Embryo and oocyte outcomes.

**Treatment arm**	**Placebo** **N = 40**	**Melatonin** **2 mg bd** **N = 41**	**Melatonin** **4 mg bd** **N = 39**	**Melatonin** **8 mg bd** **N = 40**	**P-value^a^**	**IRR** **(95% CI)^b^**	**P-value for** **trend across** **groups^b^**	**Any Melatonin** **N = 120**	**P-value^c^**
Number of oocytes	8.0 (3.25–13.0)	9.0 (0.0–16.0)	6.0 (0.0–14.0)	7.5 (3.25–11.0)	0.8^a^	0.97 (0.92, 1.01)	0.2	8.0 (3.0–14.0)	0.4^c^
Number of fertilized oocytes	3.0 (1.0–6.0)	4.0 (0.0–7.5)	2.0 (0.0–5.0)	4.0 (1.25–6.0)	0.6^a^	1.00 (0.93, 1.07)	0.9	4.0 (1.0–7.0)	0.8^c^
Number of embryos	3.0 (1.0–5.75)	4.0 (0.0–7.5)	2.0 (0.0–5.0)	4.0 (1.25–6.0)	0.6^a^	1.00 (0.93, 1.07)	0.9	3.0 (0.0–7.0)	0.9^c^
Number of embryos utilized	2.0 (0.25–3.0)	1.0 (0.0–3.0)	1.0 (0.0–2.0)	2.0 (0.25–3.0)	0.7^a^	0.94 (0.85, 1.03)	0.2	2.0 (0.0–3.0)	0.4^c^
Number of good embryos (A and B)	1.5 (0.0–3.75)	2.0 (0.0–5.0)	2.0 (0.0–4.0)	2.0 (0.0–3.75)	0.9^a^	1.00 (0.91, 1.07)	0.7	2.0 (0.0–4.0)	0.8^c^
Number of poor embryos (C D and X)	1.0 (0.0–2.0)	0.0 (0.0–3.0)	0.0 (0.0–1.0)	1.0 (0.0–3.0)	0.1^a^	1.03 (0.91, 1.05)	0.7	0.5 (0.0–0.5)	0.4^c^
Proportion of good embryos transferred (A and B) (%)	19/27 (70.4)	21/27 (77.8)	17/20 (85.0)	19/27 (70.4)	0.6^d^	–	–	57/74 (77.0)	0.5^d^

a Kruskal–Wallis.

b Poisson regression.

c Mann–Whitney U.

d *Chi-square, comparison between placebo and any melatonin; Results reported as Median (IQR) unless otherwise stated; IRR: incidence rate ratio; Canceled cycles considered as having zero oocytes and embryos; X = Embryos that were created but were discarded by Day 3*.

There were no statistically significant differences in median number of embryos or quality of embryos across all groups or when comparing placebo with any dose of melatonin (Table 3).

### Clinical pregnancy, live birth, miscarriage, adverse events

All clinical pregnancies resulted in a live birth. Neither the clinical pregnancy/live birth rate (p = 0.7) nor the rate of canceled cycles before OPU (p = 0.3) showed a dose-response relationship between groups (Table 4). There were no clinical pregnancies in the 30 women aged ≥40 years. The clinical pregnancy rate for all the women who took melatonin (all three groups combined) was higher than those taking a placebo, but this did not reach statistical significance [21.7 vs. 15.0%, OR 1.57 (95% CI 0.59, 4.14), p = 0.4, absolute risk reduction (ARR) 6.7% (95% CI −6.6, 20.0%)]. This result did not differ significantly in the PP analysis (22.8 vs. 16.7%, OR 1.48 (95% CI 0.56, 3.94), p = 0.4, ARR 6.1% (95% CI −8.3, 20.5%)]. We performed a logistic regression, including age as the only covariate found to effect clinical pregnancy rate in a univariate analysis, and this did not change the result significantly [adjusted OR 1.73 (95% CI 0.62, 4.78, p = 0.3)].

**Table 4:** Clinical outcomes.

	**Placebo BD** **N = 40**	**Melatonin 2 mg BD** **N = 41**	**Melatonin 4 mg BD** **N = 39**	**Melatonin 8 mg BD** **N = 40**	**P-value**	**P-value** **for trend** **across groups**	**Any melatonin** **N = 120**	**OR** **(95% CI)**	**P-value^c^**
Canceled cycle before OPU (%)	6/40 (15.0)	12/41 (29.3)	10/39 (25.6)	6/40 (15.0)	0.3^a^	0.3^b^	28/120 (23.3)	1.73 (0.66, 4.53)	0.3
Canceled cycle between OPU and ET (%)	7/40 (17.5)	2/41 (4.9)	9/39 (23.1)	7/40 (17.5)	0.1^a^	0.5^b^	18/120 (15.8)	0.83 (0.32, 2.17)	0.6
Biochemical pregnancy rate per cycle started (%)	8/40 (20.0)	14/41 (34.1)	7/39 (17.9)	9/40 (22.5)	0.3^a^	0.8^b^	30/120 (25.0)	1.33 (0.55, 3.21)	0.5
CPR per cycle started (%)	6/40 (15.0)	11/41 (26.8)	6/39 (15.4)	9/40 (22.5)	0.5^a^	0.7^b^	26/120 (21.7)	1.57 (0.59, 4.14)	0.4
CPR per ET (%)	6/27 (22.2)	11/27 (40.7)	6/20 (30.0)	9/27 (33.3)	0.5^a^	0.6^b^	26/74 (35.1)	1.90 (0.68–5.29)	0.2
CPR per ET^*^g (%)	8/29 (27.6)	11/28 (39.3)	9/25 (36.0)	9/30 (30.0)	0.8^a^	0.9^b^	29/83 (34.9)	1.75 (0.64–4.81)	0.3
LBR per cycle started (%)	6/40 (15.0)	11/41 (26.8)	6/39 (15.4)	9/40 (22.5)	0.5^a^	0.7^b^	26/120 (21.7)	1.57 (0.59, 4.14)	0.4
LBR per ET (%)	6/27 (22.2)	11/27 (40.7)	6/20 (30.0)	9/27 (33.3)	0.5^a^	0.7^b^	26/74 (35.1)	1.90 (0.68–5.29)	0.2
LBR per ET^*^ (%)	8/29 (27.6)	11/28 (39.3)	9/25 (36.0)	9/30 (30.0)	0.8^a^	0.9 ^b^	29/83 (34.9)	1.75 (0.64–4.81)	0.3
Mean birthweight (SD)	3,240 (228)	3,249 (168)	3,267 (227)	3,492 (186)	0.8^d^	0.6^e^	3337 (342)	1.00 (0.99, 1.00)	0.7^d^
Gestation ≥ 37 weeks	5 (83.3)	10 (90.9)	6 (100.0)	8 (88.9)	0.6^a^	0.8^b^	24 (92.3)	2.40 (0.18, 31.88)	0.5

* These include the first frozen embryo transfer of patients having an elective “freeze all” cycle.

a Chi-square.

b Chi-square for trend across treatment groups.

c Chi-square, comparison between placebo and any melatonin.

d Estimated marginal means.

e Spearman-rank correlation coefficient = 0.1.

Patients who were randomized but did not take trial medication were coded as “canceled cycle before OPU.”

Of all 160 patients who were randomized, 59 (36.9%) did not reach ET in their first cycle. Ten (17%) were withdrawn prior to commencing trial medication, 20 (34%) had poor ovarian response to stimulation, 14 (24%) were canceled after OPU but before ET because of a lack of transferable embryos, 11 (19%) required a “freeze all” cycle, 2 (3.4%) had a premature LH surge and 2 (3.4%) experienced an error in stimulation medication administration. For the ITT analysis, patients who were recruited but withdrawn were coded as “canceled before OPU.” Therefore, the cancelation before OPU rate in the women taking melatonin (all doses) was 23.3% compared with 15.0% for those taking placebo [OR 1.73 (95% CI 0.66, 4.53), p = 0.3]. In the PP analysis, the canceled cycle before OPU rate was 19.3% in the any melatonin group, compared with 5.6% in the placebo group [OR 4.07 (95% CI 0.91, 18.22), p = 0.07].

The rate of miscarriage did not differ between groups, although the total number of miscarriages was small (n = 5). There were no ectopic pregnancies. One woman, in the 2 mg bd melatonin group, had a term livebirth of a baby with an absent right kidney. One baby from the placebo group was born weighing 1,300 g at 29 weeks and no babies in the melatonin groups were born <2,500 g. One patient each in the 2 and the 8 mg bd melatonin groups gave birth between 34 and 37 weeks. One patient each in the 4 and the 8 mg bd melatonin groups were diagnosed with preeclampsia and there was one case of major placenta praevia in the 2 mg bd melatonin group.

No “minor” adverse effects were reported by 33.3% of women in the placebo group and by 28.1% in the melatonin groups (p = 0.6). The commonest reported adverse effect was headache, reported by 50% of women in the placebo group and by 45% of those taking any dose of melatonin (p = 0.6). The rates of fatigue also did not differ between placebo and melatonin arms (16.7 vs. 28.1%, p = 0.2). A detailed analysis of sleep outcomes is presented elsewhere (21).

### Subanalyses

We performed a PP analysis excluding those with cancelations before OPU. There were no differences in total oocytes retrieved, oocytes fertilized, total number of embryos or utilized embryos. While there was again a trend toward an increase in both clinical pregnancy and live birth rate, this did not reach statistical significance [28.3 vs. 17.6%, OR 1.84 (95% CI 0.68, 4.96), p = 0.2]. When also excluding those with cycles canceled after a successful OPU but before ET (for reasons other than a “freeze all” cycle) there remained no statistically significant increase in clinical pregnancy or live birth rate [31.3 vs. 18.8%, OR 1.98 (95% CI 0.73, 5.38), p = 0.2].

## Discussion

We designed this clinical trial to determine the benefit of melatonin on ART outcomes and to inform sample size calculations and a target melatonin dose for future clinical trials. We found no statistically significant difference in clinical pregnancy rates in women taking melatonin during their stimulation cycle.

Our interest in melatonin as a method of improving ART success was primarily due to its potent effects as an anti-oxidant, as opposed to any presumed effects on sleep. Melatonin is unique in the family of anti-oxidants for several reasons. One of its most important properties is that it is a suicidal terminal anti-oxidant. This means that, unlike other anti-oxidants, under no circumstances does melatonin act as an oxidant and all of its metabolites are either also anti-oxidants or are stable (22). In addition, it is amphiphilic, allowing it to gain access to intra- and extra-cellular targets; it acts via receptors but also directly on free radicals; and it has the ability to potentiate the actions of other endogenous anti-oxidants (3). Melatonin has a relatively short half-life (16). It is likely that a single daily dosing regimen, such as those tested previously, would not achieve sustained anti-oxidant effects during the IVF cycle. This may, at least in part, explain why previous studies exploring the efficacy of melatonin in improving IVF success have been inconclusive (23). For this reason, we chose to examine a more frequent dosing regimen, twice per day, together with higher doses (16).

### FF melatonin and sex steroids

Serum and FF concentrations of melatonin and sex steroids were measured so that we would be able to assess changes in relation to primary and secondary outcomes. Our dosing regimens resulted in measurable differences in concentrations of circulating and FF melatonin. In that regard, we observed an increase in FF concentrations of melatonin of three-, six-, nine-fold for our three doses, compared with placebo. The absolute concentrations are dissimilar to those observed by Tamura and colleagues, however, the relative increase compared with controls was similar between our studies (three- vs. four-fold) (2). Therefore, the difference in absolute concentrations are most likely a reflection of the different assay techniques used.

There was no difference in FF concentrations of estradiol and progesterone between the placebo and melatonin groups, suggesting that melatonin is not significantly associated with the synthesis of these hormones in the follicle. This is in contrast to previous findings in swine models (24).

### Oocyte and embryo outcomes

Based on the findings of previous reports, we also assessed the impact of melatonin on oocyte and embryo number and morphology. We found no apparent effects on these outcomes.

In a recent retrospective analysis, Tong et al. (25) identified that higher endogenous FF melatonin concentrations are correlated with a higher response to ovarian stimulation. While a moderate correlation existed, the lack of molecular pathway analysis or controlling for other factors associated with ovarian response makes it difficult to conclude that melatonin deficiency causes poor ovarian response.

Our findings also contrast with those of others (3, 4, 11, 12). Eryilmaz and colleagues studied 60 women with sleep disorders and unexplained infertility (11). They found that a single 3 mg dose of melatonin given at night almost doubled the number of oocytes retrieved, from 6.9 to 11.5 (p < 0.001) and the number of MII oocytes (4.0–9.0, p < 0.001), and increased the proportion of grade 1 embryos from 45 to 69% (p = 0.05). However, while that trial was randomized it was not blinded and introduced further bias by excluding canceled cycles from analysis. The authors were also unable to control for possible confounding factors such as number of previous failed IVF cycles and parity. Another study that used patients as their own controls after failing a non-melatonin treated IVF cycle also found an increase in the proportions of fertilized oocytes and good quality embryos (12). The within-subjects design limits the strength of the conclusions of this study because of the phenomenon of regression to the mean (26).

In keeping with our findings, Batiǒglu et al. (4) found that, when comparing controls with melatonin, the total number of oocytes retrieved and the number of mature oocytes did not differ (10.9 ± 4.0 vs. 12.0 ± 6.0, p = 0.14). This study excluded canceled cycles. In our study, we focussed on first cycles, controlled for multiple demographic variables, and were able to assess multiple doses of melatonin. In our sub-analysis, after excluding patients who had their cycles canceled before OPU, there was no significant difference between groups in any oocyte or embryo parameters, although the difference widened. It is therefore probable that previous reports of improvements in embryological outcomes after melatonin treatment that have excluded canceled cycles have overestimated the benefit of melatonin in these circumstances. Our inability to confirm measurable effects on gametes and embryos with any dose casts significant doubt on the plausible mechanisms by which melatonin might improve live birth rates.

### Clinical pregnancy, live birth rates, and adverse events

Ours is the first study to assess the live birth rate in women taking melatonin during their first IVF cycle. We were able to demonstrate a statistically significant dose-dependent increase in FF melatonin concentrations (nine-fold for 8 mg bd group) and while this resulted in a trend toward an increase in clinical pregnancy rate (and live birth rate) when all doses were compared with placebo, this did not reach statistical significance. When considered together with the absence of any measurable effects on egg or embryo number or quality and with previous studies that have also failed to show an improvement in pregnancy rate (2, 4, 11) it does cast doubt on the potential efficacy of melatonin in this context. A recently published randomized trial assessing the combined use of melatonin with myo-inositol specifically in participants with polycystic ovarian syndrome (PCOS) has also found no significant improvement in clinical pregnancy rate (27).

Despite significant increases in serum melatonin concentrations, the rate of “minor” side-effects was very low and comparable between placebo and melatonin groups. There were absolute differences in the reported rates of fatigue, however, this did not reach statistical significance, likely because of the small sample involved. Despite this, in a separately published detailed analysis of sleep outcomes from this trial, there were no differences in daytime sleepiness or night time sleep quality between the groups (21).

### Canceled cycles

The number of recruited women who did not reach fresh ET was 59 (36.9%). This was largely due to the number of canceled cycles before OPU. The clinic-specified cancelation criteria (<3 follicles >17 mm or risk of severe OHSS) may have contributed to the high cancelation rate seen in this study. Interestingly, in our PP analysis (excluding women who were withdrawn), there was a trend toward an increase in canceled cycles before OPU when comparing any dose of melatonin with placebo, although this also did not reach statistical significance [19.3 vs. 5.6%, OR 4.07 (95% CI 0.91, 18.22), p = 0.07]. This result needs to be interpreted with caution because of the wide confidence interval and small sample size. Nevertheless, we cannot exclude the possibility that melatonin may cause a reduction in the success of ovarian stimulation.

### Limitations and generalizability

This is the first dose-finding double-blind placebo-controlled randomized trial assessing the effect of melatonin on live birth rates in women undergoing their first IVF cycle. A further key strength is the analysis of circulating and FF melatonin concentrations following the administration of three doses of melatonin administered twice daily to improve sustained levels.

While this study showed no clear evidence for an improvement in pregnancy rates, a suggestive trend encourages us to consider a definitive study with livebirth productivity rate as the appropriate measure.

In addition, as we sought to determine the outcome of the first cycle only, we were unable to determine the outcomes of embryos that were frozen from a trial cycle. This, therefore, does not account for cumulative pregnancy rates from a melatonin treatment cycle. However, as some of these embryos may never be used, including embryo outcomes from only those that are will significantly bias the outcome analysis. This may be partially addressed by determining a time-dependent end-point at which to calculate cumulative pregnancy rates. We plan to undertake this analysis in the future, once data from all frozen transfers becomes available.

It can also be argued that the maximal benefit of an adjuvant therapy such as melatonin would be experienced by patients who are “poor responders.” Our study focussed on women having their first cycle of IVF and therefore, our findings cannot be applied to a general IVF population, where women have previously had multiple unsuccessful cycles of IVF. In order to determine whether melatonin had a positive effect in a “poor responder” population, a RCT in this specific selected population would need to be conducted. Finally, the clinical pregnancy rate in our placebo group may appear to be low (15%) but the confidence interval around this point estimate is wide (95% CI 5.7%, 29.8%). In order to ensure that this relatively low success rate was not a reflection of selection bias, we assessed the intention-to-treat clinical pregnancy outcomes of women who had declined participation in the trial (who therefore, were eligible for inclusion and comparable to the placebo group). In this population of 211 women, the clinical pregnancy rate was 16% per cycle started. Sixty-four percent reached embryo transfer (compared with 63% in the MIART placebo group) and 12% had a canceled cycle before oocyte retrieval (compared with 15% in MIART placebo group). Reassuringly, these results are comparable to the MIART placebo group.

### Interpretation

In this clinical trial, we have shown that oral melatonin in high doses can result in supraphysiological serum and FF concentrations. In addition, no statistically significant improvement in clinical pregnancy rate was found. Furthermore, because of the lack of difference in secondary outcomes (including oocyte and embryo parameters), and no dose dependent effect, this finding should be interpreted with caution. In order to confirm this potential improvement in clinical pregnancy rate, a much larger study [sample size of ~1,500 patients (allowing for withdrawals) with a 1:1 allocation ratio comparing placebo with melatonin, α = 0.05 to detect a difference similar to that found in our study] would need to be conducted. Recognizing that our study lacked power for the primary outcome, the observed effect size in this pilot dose-finding study could inform the design of a larger randomized controlled trial of melatonin in IVF. Based on both biochemical and clinical effects identified in our trial, such a study would focus on a dose of 4–8 mg twice per day (as opposed to the previously investigated dose of 3–4 mg once per day).

Hence, as we have demonstrated no differences in oocyte or embryo outcomes and no dose-dependent clinical effects, the plausibility of a positive effect of melatonin on clinical pregnancy rate remains questionable until the findings in our trial can be replicated in further larger RCTs.

## Author contributions

SF, LR, and EW conceived the idea for the study and led study design, implementation and data collection. They also were responsible for drafting of the paper and review of the final manuscript. SF and LR were responsible for recruitment. BV, NL, NH, MW, ML, AL, CR, PT, and KL contributed significantly to recruitment and the drafting and approval of the final manuscript.

### Conflict of interest statement

LR is a Minority shareholder in Monash IVF Group, has unrestricted grants from MSD®, Merck-Serono®, and Ferring® and receives consulting fees from Ferring®. The remaining authors declare that the research was conducted in the absence of any commercial or financial relationships that could be construed as a potential conflict of interest.
